# Applicability of an active matrix metalloproteinase-8 point-of-care test in an oral and maxillofacial surgery clinic: a pilot study

**DOI:** 10.1007/s10266-023-00821-0

**Published:** 2023-05-30

**Authors:** Essi Kallio, Tero Puolakkainen, Taina Tervahartiala, Johanna Snäll, Emilia Marttila, Timo Sorsa, Johanna Uittamo

**Affiliations:** 1 Department of Oral and Maxillofacial Diseases, University of Helsinki and Helsinki University Hospital, Biomedicum 1, Haartmaninkatu 8 (PL 63), 00014 Helsinki, Finland; 2 Department of Oral Diseases, Karolinska Institutet, Huddinge, Sweden

**Keywords:** aMMP-8, Metalloproteinase, Infection, Pilot study, Oral and maxillofacial surgery

## Abstract

**Objectives:**

Matrix metalloproteinases are enzymes that participate in numerous inflammatory responses and have been targeted as biomarkers in numerous pathologic states. The detection of active matrix metalloproteinase-8 (aMMP-8) using a mouthrinse point-of-care test (POCT) has emerged as a diagnostic marker for periodontitis and other systemic inflammatory states. The objective of this pilot study was to assess the applicability of aMMP-8 POCT in an oral and maxillofacial surgery clinic and to evaluate the relationship between aMMP-8 levels and different patient groups.

**Materials and methods:**

aMMP-8 POCT samples were collected from patients in an oral and maxillofacial surgery clinic during a one-month period. aMMP-8 levels were analyzed using a chairside lateral-flow immunotest and a digital reader. Clinically relevant patient variables were collected and descriptively evaluated. aMMP-8 levels over 20 ng/ml were considered to be elevated.

**Results:**

A total of 115 patients were interviewed of which 112 agreed to the test (97.4%). Elevated aMMP-8 levels were observed in 58 (51.8%) patients. Bone loss was noted in 75 (67.0%) patients. Of these patients, aMMP-8 levels were elevated in 47 (62.7%) patients. Patients at an increased risk of infection had 35.5% higher aMMP-8 values on average compared to patients with no prior illnesses.

**Conclusion:**

aMMP-8 POCT provides a non-invasive and reliable method for measuring aMMP-8 levels. Future studies are warranted to assess the clinical relevance between elevated aMMP-8 levels and specific patient groups.

**Clinical relevance:**

The rapid availability of the test score allows an immediate impact on treatment planning.

## Introduction

The orofacial region is susceptible to a number of pathologic states including benign and malignant lesions, conditions and deformities of the facial bones, craniofacial trauma as well as head and neck infections [1–3]. Despite standard of care and evidence-based treatment protocols, patients of these groups are at risk of sustaining postoperative issues often due to idiopathic reasons. For example, patients undergoing orthognathic surgery or requiring surgical fixation for mandibular fractures are at a significant risk of sustaining postoperative infections [4–6]. These infections are linked to increased hospital-stay and burden patients with pain and periods of unproductivity [7]. Importantly, the incidence rates of specific infections requiring intensive care are alarmingly increasing [8]. Therefore, there is an urgent need for better diagnostic tools to investigate hidden risk factors, and to identify which patients are predisposed to postoperative complications.

Matrix metalloproteinases (MMPs) are a family of proteolytic enzymes that have paramount roles in a myriad of physiologic and pathologic events including cell differentiation, organogenesis, and apoptosis [9]. Different MMPs have also been shown to pertain to states of various malignancies and inflammation as measures of tissue destruction [10]. Especially elevated levels of active MMP-8 (aMMP-8) based on local analyses have emerged as diagnostic enzymes for inflammatory states such as periodontitis, peri-implantitis and type II diabetes [11–13]. In addition, local analysis of elevated MMP-8 levels has recently been linked with higher risk for severe COVID-infections [14, 15].

The detection of aMMP-8 using a mouth rinse point-of-care test (POCT) has been established as a safe, non-invasive and reliable screening tool for diagnosing these pathologic states [14, 16, 17]. Recent findings have also linked elevated aMMP-8 levels to other morbid states including pancreatitis as well as head and neck cancer [18, 19]. As these findings are novel, there is a paucity of studies assessing the applicability of aMMP-8 as a clinically relevant enzyme serving as a biomarker in predicting postoperative infection rates. Therefore, these studies encourage and evince the need for prospective clinical studies aiming to elucidate the applicability aMMP-8 as a clinical point-of-care diagnostic enzyme in the field of oral and maxillofacial surgery.

Based on the aforementioned assertions, we hypothesize that elevated aMMP-8 levels may be linked to specific oral and maxillofacial surgery patient populations. In this pilot study, the primary aim was to evaluate the applicability of measuring aMMP-8 levels using a POCT in an oral and maxillofacial surgery clinic.

## Materials and methods

### Study design

A prospective study was conducted for patients aged 18 years or older prior to inpatient and/or outpatient surgical procedures in the Helsinki University Hospital (HUH) oral and maxillofacial surgery clinic. These patients composed the population from which cohorts were recruited during a 1-month period in June 2021. HUH treats patients with a catchment area of nearly 2 million inhabitants.

The primary inclusion criteria for recruitment were age (any patient aged 18 years or older) and admittance to the care of the oral and maxillofacial surgery service. Exclusion criteria included patients under the age of 18 years and patients who did not agree to the POCT.

### Study methods

Patients’ infection risk and periodontal status was evaluated and compared to corresponding aMMP-8 levels measured with POCT. The applicability of the test was evaluated in this specific patient population. Recruited patients were also interviewed for the medical history and use of tobacco and alcohol products.

### Study variables

Patients were classified by their medical status and background into three groups. “No prior illness” included healthy patients without any diagnosed general or systemic conditions. “Increased risk of infection” included patients with immunosuppressive status or increased risk for sustain infections such as previous chemo- or radiotherapy, type I and II diabetes, recipient of an organ transplant, any type of autoimmune disorder, liver failure, and cancer. “Other” included patients with medical conditions which do not directly increase risk of infection such as increased blood pressure, neurological disorders, and atherosclerosis.

Smoking history was listed as pack years, and alcohol consumption was classified in to four risk categories (no risk, low risk, moderate risk and heavy risk). Alcohol consumption dose limits for women; 0–1 servings per day (low), 7 per week (moderate), 12–16 per week or more (heavy). For men: 0–2 servings per day (low), 14 per week (moderate), 23–24 per week or more (heavy) [20].

Alveolar bone loss was evaluated based on periodontal inspection and available X-rays. The periodontal inspection was based on the Community Periodontal Index (CPI), and periodontal pocket depths were measured around teeth from all six sextants using a standard millimetre probe. Loss of gingival attachment of 4 mm or more was defined as alveolar bone loss. Bone loss (defined as either vertical or horizontal bone loss as well as periapical lesions) was also evaluated from available patient radiographs that were no more than 6 months old.

Mouthrinse sample collection and execution of aMMP-8 POCT was done immediately after the interview and before the surgical procedure.

### Periosafe protocol

The protocol for the Periosafe immunotest has been described in detail previously. [17] Mouthrinse samples were gathered by having patients rinse their mouth with a water-based medium for 1 min, which was subsequently collected and analyzed using a chairside lateral-flow immunotest, PerioSafe, and digital reader, ORALyzer, apparatus according to the manufacturer’s instructions. After the aMMP-8 POCT, all collected mouthrinse samples were frozen and stored in − 20 °C freezers. The lateral-flow sandwich immunoassay is based on the utilization of monoclonal antibodies conjugated to latex particles. [21] Three drops of the collected mouthrinse sample were placed onto the reader and results were available in five minutes. Results were presented in an immunochromatographic and numerical manner with the ORALyzer reader. aMMP-8 levels of 20 ng/ml and over were considered to be elevated. The qualitative result was visible as one or two lines on the test stick; a single line indicating aMMP-8 level less than 20 ng/ml (non-elevated); and two lines as aMMP-8 level 20 ng/ml or more (elevated). The visible result on each test stick was documented by photography.

### Ethical considerations

The study design has been by approved the Helsinki University Hospital Internal Review Board (HUS/126/2021) and the Local Ethical Committee (HUS/1309/2021) in accordance with the Helsinki declaration. Written consent was received from all patients recruited to the study.

## Results

A total of 115 patients were interviewed of which 112 (97.4%) agreed to the aMMP-8 POCT providing a high rate of consent. The mean age of the included patients was 56.7 years. Increased levels of aMMP-8 concentrations (≥ 20 ng/ml) were observed in 58 (51.8%) of the included patients. (Table 1) Additionally, it was noted that due to the simplicity of the test, it could also be performed by other members of the treatment personnel in addition to dentists and physicians. aMMP-8 values were compared to clinical and radiographic findings of periodontal bone loss (Table 2). Bone loss based on CPI and previous radiographic images was noted in 75 (67.0%) patients. Of these patients, aMMP-8 levels measured by the POCT were elevated in 47 (62.7%) patients. Interestingly, elevated aMMP-8 levels were observed in 11 (28.2%) patients with no prior periodontal bone loss.

**Table 1:** Descriptive statistics and background of patients with normal or increased aMMP-8 levels

Variable	aMMP-8 (≥ 20 ng/ml)			aMMP-8 (< 20 ng/ml)		
	No. of patients	%	% of *n*	No. of patients	%	% of *n*
Total	58	51.8		54	48.2	
Age						
Mean	60.0			53.3		
Range	20.3–89.2			19.2–93.3		
Sex						
Male	32	55.2	61.5	20	37.0	38.5
Female	26	44.8	43.3	34	63.0	56.7
Smoking						
No	25	43.1	47.2	28	51.9	52.8
Yes	15	25.9	35.7	9	16.7	64.3
Former	18	31.0	51.4	17	31.5	48.6
Alcohol						
No risk	25	43.1	49.0	26	48.1	51.0
Low risk	26	44.8	49.1	27	50.0	50.9
Moderate risk	5	8.6	83.3	1	1.9	16.7
High risk	2	3.4	100.0	0	0.0	0.0

**Table 2:** Relationship between periodontal bone loss and a-MMP8 levels

Variable	aMMP-8 (≥ 20 ng/ml)			aMMP-8 (< 20 ng/ml)		
	No. of patients	%	% of *n*	No. of patients	%	% of *n*
Periodontal bone loss						
No	11	19.0	29.7	26	48.1	70.3
Yes	47	81.0	62.7	28	51.9	37.3

**Fig. 1:**
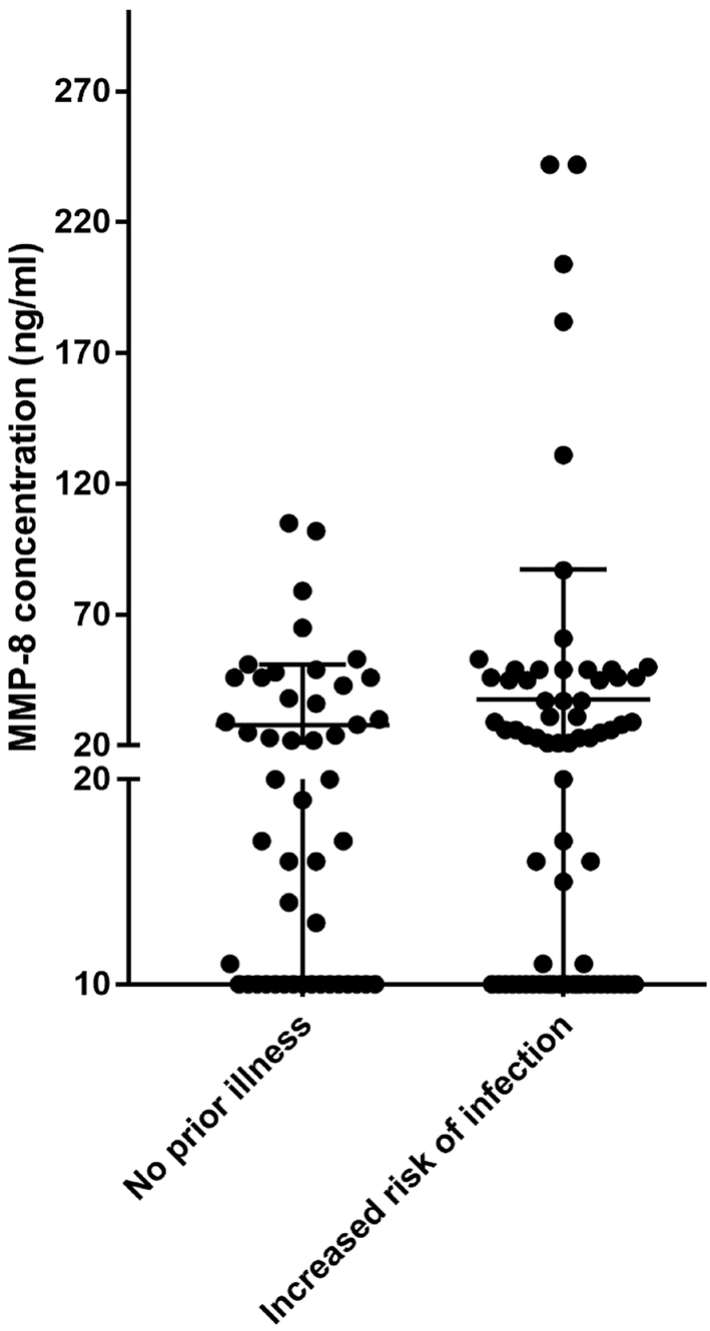
Comparison of aMMP-8 level range between patients with no prior illness and patients with medical background proning them to an increased risk of infection. A trend in elevated levels of aMMP-8 concentration in patients with a background of increased risk of infection was observed compared to healthy patients with no prior illness

Figure 1 displays a scatter chart of measured aMMP-8 levels in patients who had either no prior medical condition or an increased risk of infection. A trend was seen between medical status and aMMP-8 levels as patients at an increased risk of infection had 35.5% higher aMMP-8 values on average compared to patients with no prior illnesses (37.6 ± 49,58 ng/ml vs 27.8 ± 23.3 ng/ml, *p* = 0.15).

**Fig. 2:**
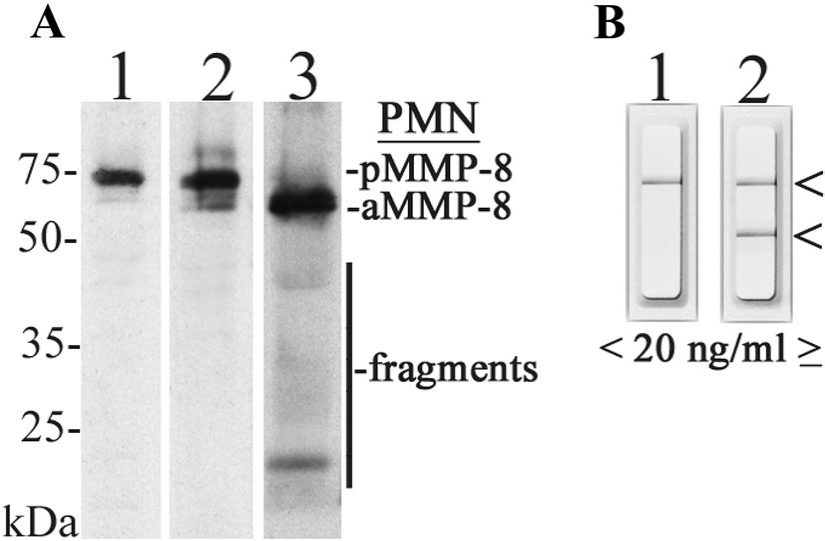
Representative western immunoblot for molecular forms and species of MMP-8/collagenase-2 in the studied mouthrinse samples. **A** Lane 1: recombinant human MMP-8 (100 ng), monoclonal antibody; lane 2: mouthrinse sample of systemically and healthy subject, monoclonal antibody; lane 3: mouthrinse sample of systemically and orally diseased subject, monoclonal antibody. PMN indicates polymorphonuclear leukocyte; pMMP-8 indicates latent proMMP-8, aMMP-8 indicates active MMP-8, and fragments indicate lower (50 kDa) molecular size MMP-8 species due the activation and related fragmentation. **B** Negative (−, one line, 20 ng/ml aMMP-8, lane 1) and positive (+, two lines, ≥ 20 ng/ml aMMP-8, lane 2) chair-side (PoC) lateral-flow immunotest outcomes indicated by arrows on the right

Representative western immunoblot analysis of MMP-8 in the studied mouthrinse samples from orally and systemically healthy and diseased study subjects are shown in the Fig. 2. MMP-8 was in latent proform in healthy samples (Fig. 2A, lane 2, Fig. 2B, lane 1) and converted to active and fragmented forms in diseased samples (Fig. 2A, lane 3, Fig. 2B, lane 2) as analyzed by monoclonal anti-MMP-8 antibody.

## Discussion

Elevated aMMP-8 but not total MMP-8 levels in mouthrinse and saliva recently have been shown to predict the clinical manifestation of systemic inflammatory states such as type II diabetes [22]. Therefore, aMMP-8 levels could be a critical regulator of inflammation, tissue destruction and healing, and aMMP-8 levels potentially could be used to predict postoperative infection risks. In this pilot study, we were able to show that aMMP-8 POCT could easily be integrated to the examination and treatment planning of patients undergoing procedures related to oral and maxillofacial surgery. Additionally, the test could also be performed by other members of the treatment personnel in addition to dentists and physicians.

The development of non-invasive diagnostics tests has led to a substantial increase of interest in research communities aiming to measure and assess correlations between biological enzymes and mediators related to different pathological states. However, the applicability of measuring biomarkers in clinical settings remains somewhat limited especially due to issues with sensitivity and specificity, and their roles seemingly remain secondary to other diagnostic measures. Measurement of aMMP-8 levels using a POCT has emerged as an auspicious method to reliably evaluate active periodontitis [23–25]. As the results of this pilot study support the application of aMMP-8 POCT in clinical practice in an oral and maxillofacial surgery clinic, future studies are directed toward the clinical significance of elevated aMMP-8 levels to predict postoperative healing and rates of infection these patients may be subjected to.

One of the most viable factors used to evaluate the applicability of a diagnostic test in clinical practice is the rate of consent. For this pilot study, the rate of consent of the interviewed patients to participate in this pilot study was nearly 100%. Importantly, the non-invasive nature of the test did not interrupt, impede or modify the patient’s allocated treatment plan in any way. Additionally, the rapid availability of the test score (5–10 min chair-/bed-side) allows an almost immediate impact on treatment planning without the necessary handling time associated with measuring blood panels. In terms of use, the aMMP-8 POCT offers a safe, non-invasive and efficient way of measuring enzymes and diagnostic enzymes of clinical interest and easily can be integrated into a standard examination and diagnostic protocol.

Bone loss was effectively detected by the POCT. Of patients with elevated aMMP-8 levels, 81.0% had some degree of periodontal or alveolar bone loss that could be confirmed clinically or radiographically. In turn, 37.3% of patients with periodontal bone loss remained undetected by the POCT. This can be surmised to be due to the fact that the test only detects levels of active MMP-8 i.e., the test does not recognize the non-active inflammatory status in this patient population. Interestingly, almost 30% of the included patients had increased aMMP-8 levels with no clinical or radiologic signs of periodontitis. The aMMP-8 POCT can predictively detect developing periodontitis and peri-implantitis prior to clinical or radiographic manifestations [26, 27]. Overall, this provides important insight on the notion that aMMP-8 measured from oral samples are elevated in numerous systemic diseases and conditions.

Patients potentially prone to increased risk of infections exerted an elevated tendency toward elevated aMMP-8 levels in the mouthrinse samples. In this regard, it is noteworthy that systemically elevated serum aMMP-8 have recorded to reflect mortality in severe bacteremia and sepsis [28, 29]. Additional studies are required to address the usefulness of oral fluid aMMP-8 POCT-assay to analyze systemic infections.

The main limitation concerning this study is the confined study design as only one main objective was the target of interest. Additionally, the heterogenous population in relation to the relatively small sample size does not allow the drawing of any inferences concerning the clinical significance of aMMP-8 levels in this patient group. However, as the main objective was to evaluate the applicability of this specific test, the results of the pilot study confirm the non-invasiveness, efficacy and rudimentary use of aMMP-8 POCT supporting the implementation of future studies.

## Data Availability

Data set is available upon reasonable request.
